# Baseline FIB‐4 May Be a Risk Factor of Recurrence After SBRT in Patients With HBV‐Related Small HCC

**DOI:** 10.1002/cam4.70535

**Published:** 2025-01-09

**Authors:** Zhan Zeng, Yifan Han, Wengang Li, Hongyu Chen, Ning Lin, Yanyan Yu, Xiaoyuan Xu

**Affiliations:** ^1^ Department of Infectious Diseases Peking University First Hospital Beijing China; ^2^ Department of Gastroenterology Peking University First Hospital Beijing China; ^3^ Radiation Oncology Department The Fifth Medical Center of Chinese PLA General Hospital Beijing China

**Keywords:** FIB‐4, hepatocellular carcinoma, prognostic factor, recurrence

## Abstract

**Aims:**

Exploring fibrosis index‐4 (FIB‐4)'s predictive value for HBV‐related hepatocellular carcinoma (HCC) in assessing recurrence following stereotactic body radiation therapy (SBRT) in patients with HBV‐related HCC.

**Methods:**

HBV‐related HCC patients who underwent SBRT were retrospectively enrolled from March 2012 to March 2020. Patients were divided into recurrence and non‐recurrence groups based on the HCC recurrence situation. Baseline data were collected from all patients before treatment and at 3 and 6 months after treatment, and FIB‐4 was calculated at the corresponding time points. Risk factors were selected using Cox regression. The FIB‐4 was stratified for survival analysis.

**Results:**

One hundred and fifty‐two patients were included. With a mean age of 53.5 years old, 94.1% of them had liver cirrhosis. The median recurrence‐free survival (RFS) time for recurrent patients was 17.5 months. The tumor response rate of SBRT was 94.8%. HCC recurrence rates at 12, 24, 36, 48, and 60 months were 19.7% (30/152), 38.2% (58/152), 48.0% (73/152), 52.0% (79/152), and 53.3% (81/152), respectively. Cox regression showed that baseline FIB‐4 (95% CI: 1.030 ~ 1.144, *p* = 0.002) and 3 tumor nodules (95% CI: 3.727 ~ 260.663, *p* = 0.002) are risk factors for HCC recurrence. Patients with a baseline FIB‐4 > 6.55 were at a higher risk of HCC recurrence than those with a baseline FIB‐4 < 6.55 (*p* < 0.001).

**Conclusion:**

Baseline FIB‐4 is a risk factor for recurrence after SBRT in patients with HBV‐related HCC, and the predictive threshold for FIB‐4 is higher in patients with cirrhosis. For patients who received radiotherapy, postoperative FIB‐4 levels are elevated.

## 1 Introduction

Primary liver cancer is a significant health issue affecting people worldwide. Recent data show that liver cancer has become the sixth most frequently occurring cancer in the world, as well as the third leading cause of cancer death [1]. China has the highest number of liver cancer patients in the world [2]. Hepatocellular carcinoma (HCC) accounts for the vast majority of primary liver cancer. China has a large number of patients infected with HBV, and more than 80% of HCC patients in China are infected with HBV [3]. Treatments for HCC include surgery, ablation therapy, transarterial chemoembolization (TACE), radiotherapy, targeted therapy, and immunotherapy [4]. Stereotactic body radiation therapy (SBRT) is an effective type of radiotherapy with low hepatic toxicity [5].

HCC patients experience a poor prognosis due to the high recurrence rate. The risk factors for recurrence in patients with HCC have been the focus of many researchers for a long time [6, 7, 8]. However, these factors are difficult to conveniently apply in clinical practice. Fibrosis index based on four factors (FIB‐4), calculated from four common clinical indicators, was originally developed in 2006 to evaluate the degree of liver fibrosis in patients coinfected with HCV and HIV [9]. Since then, some researchers have investigated the role of FIB‐4 in predicting the prognosis of non‐alcoholic fatty liver disease, alcohol‐related liver disease, and chronic hepatitis B [10, 11, 12]. Some studies have shown the role of FIB‐4 alone or in combination with other markers in predicting recurrence after resection of HCC [13, 14]. These studies demonstrated the role of FIB‐4 in predicting the prognosis of patients with HCC after surgical resection. Here, we investigated the role of FIB‐4 in predicting the prognosis of patients with hepatitis B virus (HBV)‐related HCC after SBRT.

## 2 Material and Methods

### 2.1 Study Populations

**FIGURE 1:**
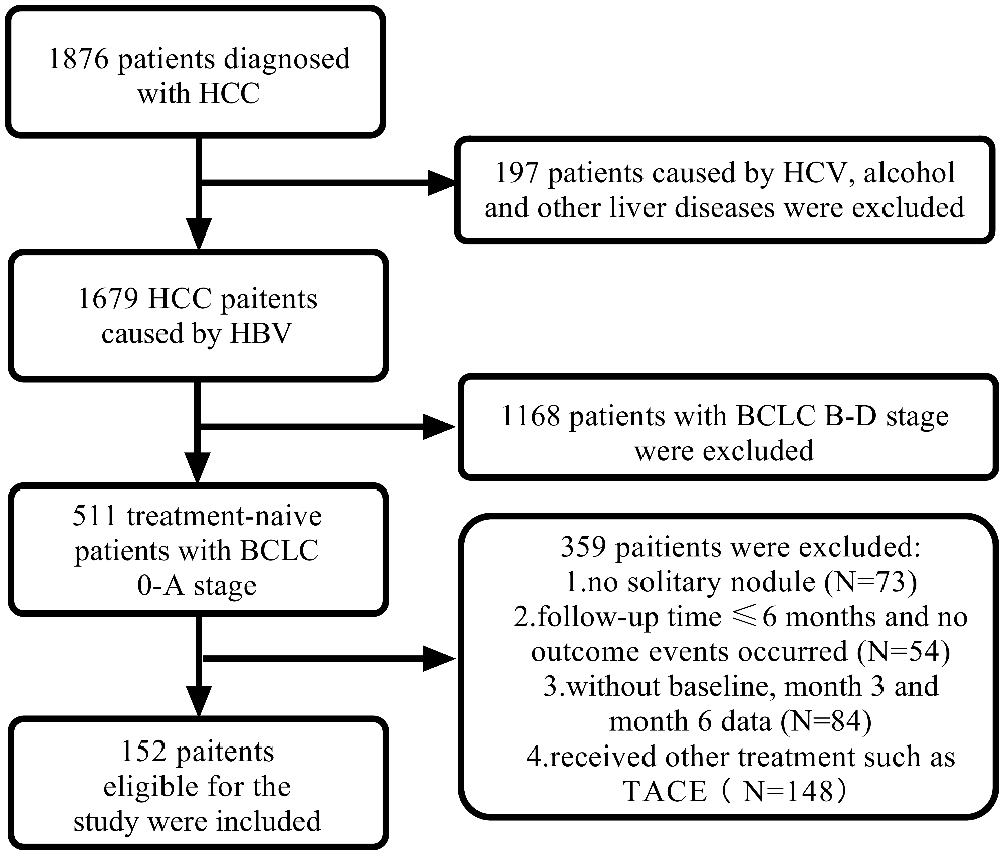
The flowchart of patients' inclusion. BCLC, Barcelona Clinic Liver Cancer; HBC, hepatitis B virus; HCC, hepatocellular carcinoma; HCV, hepatitis C virus; TACE, Transcatheter arterial chemoembolization.

The study population was obtained from the Fifth Medical Center of Chinese PLA General Hospital between March 2012 and March 2020. Inclusion criteria include: (1) Diagnosis of small HCC (single nodule < 5 cm, without microvascular infiltration, lymph node, or extrahepatic metastasis) by imaging examination or liver biopsy. (2) The individual has tested positive for Hepatitis B surface antigen for more than 6 months, with no co‐infection with hepatitis C or human immunodeficiency virus. (3) No history of long‐term alcohol consumption (duration > 5 years, ethanol intake > 40 g/d for men and > 20 g/d for women). (4) No previous treatment for HCC. (5) Child‐Pugh class A or B. (6) Barcelona Clinic Liver Cancer (BCLC) stage 0 or A. This retrospective study was approved by the Institutional Review Board of the Fifth Medical Center of the Chinese People's Liberation Army General Hospital (procedure code 2020‐063‐D) and was conducted in accordance with the Declaration of Helsinki. The flowchart was shown in Figure 1.

### 2.2 Treatment and Follow‐Up

All the patients received stereotactic body radiation therapy (SBRT) for HCC treatment. The patient was placed in the supine position and was immobilized using a positional immobilization bed and a vacuum pressure bag. One week before the SBRT treatment (CyberKnife, Accuray, USA), radiation oncologists implanted 4 to 6 fiducial markers in all the patients. These markers are placed no more than 6 cm away from the lesion. Then, the radiation oncologists delineated the planning target volume (PTV), gross tumor volume (GTV), and organs at risk (e.g., normal liver, bile duct, esophagus, duodenum, stomach, etc.). GTV refers to the size of the lesion observed in imaging examinations. PTV refers to expanding around the GTV by 3–5 mm. It encompasses 100% of the GTV while avoiding the organs at risk. All these operations were conducted using VSI CyberKnife MultiPlan (Version 4.6.1) and G4 CyberKnife Multiplan (Version 4.0.2). The normal tissue tolerance doses complied with the American Association of Physicists in Medicine (AAPM) Task Group‐101. The treatment is delivered in 5–8 fractions, with a total dosage of 48–54 Gray (Gy). The biological effective dose (BED10) ranged from 83.3 to 102.6 Gy. Fiducial tracking and dynamic respiration tracking were simultaneously utilized during the treatment process. The normal liver volume (NLV) is calculated by subtracting the gross tumor volume (GTV) from the total liver volume, ensuring that the NLV is equal to or greater than 800 cm^3^.

Follow‐up visits were required every 3 months after the treatment. Treatment efficacy was assessed at 3 months using the modified Response Evaluation Criteria in Solid Tumors (mRECIST) [15]. Tumor recurrence was assessed at 6 months. The follow‐up endpoint was recurrence, which was defined as intrahepatic recurrence, metastasis, or any form of tumor progression. Recurrence was determined through imaging and blood tests. The time from treatment to tumor recurrence was recorded as recurrence‐free survival time (RFS).

### 2.3 Data Collection

Demographic information (including age, gender, alcohol consumption, and cirrhosis) was collected at baseline. Laboratory test results were collected at baseline and at each follow‐up visit, including white blood cells (WBC), hemoglobin (HGB), platelets, alanine aminotransferase (ALT), aspartate aminotransferase (AST), albumin, total bilirubin (TBIL), and alpha‐fetoprotein (AFP). Image examination (CE‐MRI or CE‐CT) for the liver, lungs, bones, or other organs. FIB‐4 was calculated as: age × AST/(PLT × √ALT) [9].

### 2.4 Statistical Analysis

All the data related to this study were analyzed using GraphPad Prism (v8.0), IBM SPSS 26, and R (v4.3.2). Mean ± standard deviation was presented for continuous variables that conform to a normal distribution, while Median [Q1, Q3] was presented for variables that do not conform to a normal distribution. The student's t‐test was used to compare the differences between two groups in continuous and normally distributed variables. For those continuous variables that do not conform to a normal distribution, the Mann–Whitney *U* test was used for analysis. Pearson chi‐squared test and Fisher's exact test were utilized to analyze categorical variables. Spearman and Kendall rank correlation analysis was used to analyze the correlation between FIB4 and other indicators. Logistic regression was used to analyze the ability of FIB‐4 to predict in combination with other markers. Univariate and multivariate Cox regression were used to analyze the risk factors related to the recurrence of HCC. Baseline FIB‐4 cutoff values were calculated by ROC and Jordon's index, and survival analysis was performed by grouping according to the cutoff value. The Friedman test was used for the paired comparison of FIB‐4. Kaplan–Meier survival analysis was applied to different grades of FIB‐4. A significance level of *p* < 0.05 was considered statistically significant.

## 3 Result

### 3.1 Study Populations Baseline Clinical Characteristics

One hundred and fifty‐two patients who met the inclusion criteria were enrolled in the study. The mean age of all patients was 53.5 years old. 74.3% (113/152) of the patients were male, and 94.1% (143/152) of them had liver cirrhosis. The median follow‐up time for all patients was 24 months, while the median RFS time for the recurrence group was 17.5 months.

Compared with the non‐recurrence group, the recurrence group had older age (55.1 vs. 51.7 years, *p* = 0.027), higher baseline FIB‐4 (2.79 vs. 2.22, *p* = 0.038), and higher AFP (6‐month) (5.21 vs. 3.91 ng/mL, *p* = 0.017) (Table 1).

**TABLE 1:** Comparison of baseline characteristics between two groups.

Variable	Overall (*N* = 152)	Non‐recurrence (*N* = 70)	Recurrence (*N* = 82)	Statistic	*p*
Age (years)	53.5 ± 9.6	51.7 ± 9.6	55.1 ± 9.4	*t* = −2.24	0.027
Gender, *N* (%)					
Male	113 (74.34)	48 (68.57)	65 (79.27)	*χ* ^2^ = 2.27	0.132
Female	39 (25.66)	22 (31.43)	17 (20.73)		
Time (months)	24.0 [15.0, 36.0]	30.5 [24.0, 45.3]	17.5 [10.5, 26.0]	*Z* = −5.72	< 0.001
Tumor diameter (cm)	2.00 [1.50, 3.00]	1.90 [1.50, 2.60]	2.10 [1.50, 3.10]	*Z* = −1.55	0.120
Tumor num, *N* (%)					
1	143 (94.08)	68 (97.14)	75 (91.46)	—	0.289
2	8 (5.26)	2 (2.86)	6 (7.32)		
3	1 (0.66)	0 (0.00)	1 (1.22)		
BCLC, *N* (%)					
0	8 (5.26)	3 (4.29)	5 (6.10)	*χ* ^2^ = 0.02	0.893
A	144 (94.74)	67 (95.71)	77 (93.90)		
Alcohol, *N* (%)					
No	88 (57.89)	40 (57.14)	48 (58.54)	—	0.929
Yes	63 (41.45)	30 (42.86)	33 (40.24)		
No data	1 (0.66)	0 (0.00)	1 (1.22)		
Child‐Pugh, *N* (%)					
A5	109 (71.71)	51 (72.86)	58 (70.73)	*χ* ^2^ = 5.10	0.078
A6	34 (12.79)	18 (25.71)	16 (19.51)		
B	9 (5.92)	1 (1.43)	8 (9.76)		
Cirrhosis, *N* (%)					
0	9 (5.92)	5 (7.14)	4 (4.88)	*χ* ^2^ = 0.06	0.806
1	143 (94.08)	65 (92.86)	78 (95.12)		
WBC‐0 m (×10^9^/L)	4.59 ± 1.62	4.55 ± 1.62	4.62 ± 1.63	*t* = −0.25	0.806
WBC‐3 m (×10^9^/L)	3.45 ± 1.39	3.36 ± 1.30	3.53 ± 1.46	*t* = −0.74	0.464
WBC‐6 m (×10^9^/L)	3.71 ± 1.41	3.66 ± 1.41	3.76 ± 1.42	*t* = −0.43	0.671
HGB‐0 m (g/L)	141.00 [129.00, 153.50]	140.50 [130.00, 153.75]	141.00 [129.00, 152.00]	*Z* = −0.24	0.810
HGB‐3 m (g/L)	140.00 [128.00, 153.00]	141.00 [127.00, 154.00]	139.00 [128.00, 153.00]	*Z* = −0.36	0.721
HGB‐6 m (g/L)	139.00 [126.00, 152.00]	144.00 [128.00, 154.00]	139.00 [126.00, 149.00]	*Z* = −1.30	0.196
Platelets‐0 m (×10^9^/L)	120.50 [76.75, 168.50]	126.00 [80.00, 170.25]	119.00 [70.25, 164.50]	*Z* = −0.54	0.588
Platelets‐3 m (×10^9^/L)	92.50 [58.00, 130.00]	92.50 [61.30, 128.00]	92.50 [55.80, 132.00]	*Z* = −0.44	0.663
Platelets‐6 m (×10^9^/L)	96.50 [59.00, 141.00]	93.50 [59.00, 147.00]	98.00 [59.50, 130.00]	*Z* = −0.31	0.759
ALT‐0 m (U/L)	28.00 [18.75, 40.25]	27.00 [18.00, 38.00]	31.00 [19.25, 41.75]	*Z* = −0.96	0.338
ALT‐3 m (U/L)	31.00 [23.00, 42.00]	32.00 [23.00, 42.80]	30.00 [23.30, 41.80]	*Z* = −0.70	0.483
ALT‐6 m (U/L)	28.00 [22.00, 37.00]	28.00 [23.00, 38.80]	27.00 [21.00, 35.00]	*Z* = −1.73	0.083
AST‐0 m (U/L)	29.00 [22.75, 42.25]	28.50 [22.00, 34.00]	30.00 [23.00, 45.75]	*Z* = −1.56	0.118
AST‐3 m (U/L)	35.00 [30.00, 43.00]	35.00 [30.30, 42.80]	35.00 [29.00, 42.80]	*Z* = −0.17	0.865
AST‐6 m (U/L)	34.00 [25.00, 41.00]	34.00 [27.30, 40.80]	34.00 [25.00, 40.80]	*Z* = −0.31	0.756
Albumin‐0 m (g/L)	39.00 [36.00, 42.00]	39.00 [36.00, 42.75]	39.00 [36.00, 42.00]	*Z* = −0.64	0.525
Albumin‐3 m (g/L)	35.00 [30.00, 43.00]	39.00 [35.00, 43.00]	38.50 [34.00, 42.00]	*Z* = −1.26	0.209
Albumin‐6 m (g/L)	34.00 [25.00, 41.00]	40.00 [36.00, 43.00]	38.00 [34.00, 42.00]	*Z* = −1.67	0.095
TBIL‐0 m (μmol/L)	13.85 [10.60, 19.12]	13.25 [10.60, 18.05]	14.25 [10.70, 20.62]	*Z* = −0.57	0.568
TBIL‐3 m (μmol/L)	14.60 [11.30, 19.00]	13.60 [10.80, 18.30]	15.10 [11.70, 20.30]	*Z* = −1.00	0.316
TBIL‐6 m (μmol/L)	15.40 [11.90, 20.00]	15.20 [11.70, 18.20]	15.60 [12.40, 22.20]	*Z* = −1.11	0.268
AFP‐0 m (ng/mL)	13.71 [4.08, 114.20]	10.60 [3.92, 67.51]	19.37 [4.54, 186.45]	*Z* = −0.97	0.330
AFP‐3 m (ng/mL)	5.44 [3.52, 10.90]	5.12 [3.61, 7.50]	5.55 [3.46, 13.90]	*Z* = −1.16	0.248
AFP‐6 m (ng/mL)	4.48 [3.10, 7.61]	3.91 [2.98, 5.50]	5.21 [3.16, 10.30]	*Z* = −2.39	0.017
FIB‐4 (baseline)	2.58 [1.64, 4.76]	2.22 [1.54, 4.16]	2.79 [1.69, 5.78]	*Z* = −2.07	0.038
FIB‐4 (3 months)	3.86 [2.22, 6.31]	3.14 [2.12, 5.67]	4.26 [2.39, 7.73]	*Z* = −1.71	0.088
FIB‐4 (6 months)	3.54 [2.06, 6.02]	3.12 [1.96, 5.45]	3.88 [2.21, 7.25]	*Z* = −1.36	0.174

*Note: Z*: Mann–Whitney *U* test; *t*: Student's *t* test; *χ* ^2^: Pearson chi‐squared test or Fisher's exact test.

Abbreviations: AFP, alpha‐fetoprotein; ALT, alanine transaminase; AST, aspartate aminotransferase; BCLC, Barcelona Clinic Liver Cancer; FIB‐4, fibrosis index based on four factors; HGB, hemoglobin; TBIL, total bilirubin; WBC, white blood cells.

### 3.2 Toxicity, Tumor Response, and Recurrence

After SBRT, some patients in both groups had mild ALT or AST elevations, with an incidence of 26.8% in the recurrence group and 28.6% in the non‐recurrence group (*p* = 0.368). Except for this, no serious toxic side effects were observed.

**FIGURE 2:**
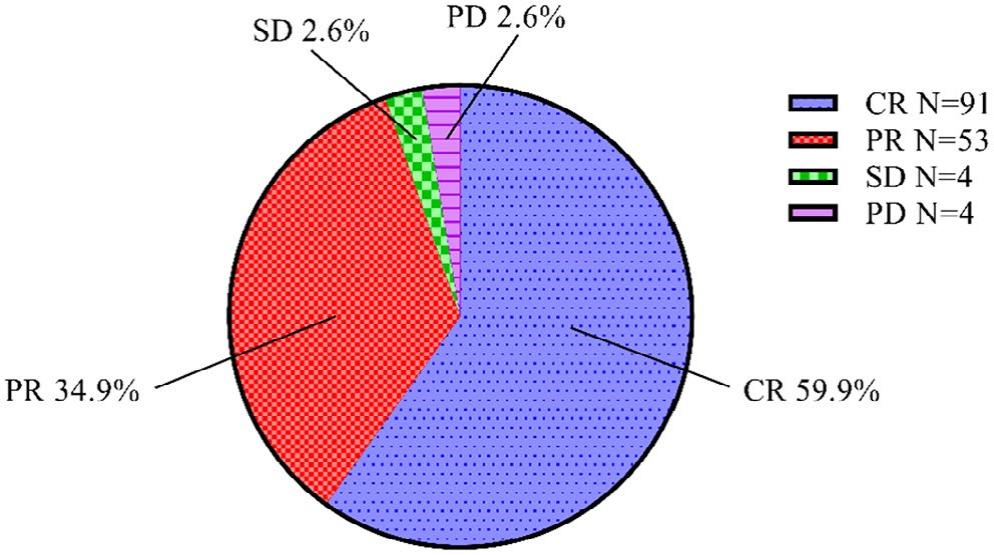
The sector graph of tumor response in month 3. CR, complete response; PD, progressive disease; PR, partial response; SD, stable disease.

At month 3 after treatment, we evaluated the treatment efficacy according to the mRECIST criteria. The overall efficacy of the treatment was 94.8%, with 59.9% (91/152) of patients achieving complete response (CR) and 34.9% (53/152) achieving partial response (PR). Both stable disease (SD) and progressive disease (PD) were observed in 2.6% (4/152) of patients (Figure 2).

**FIGURE 3:**
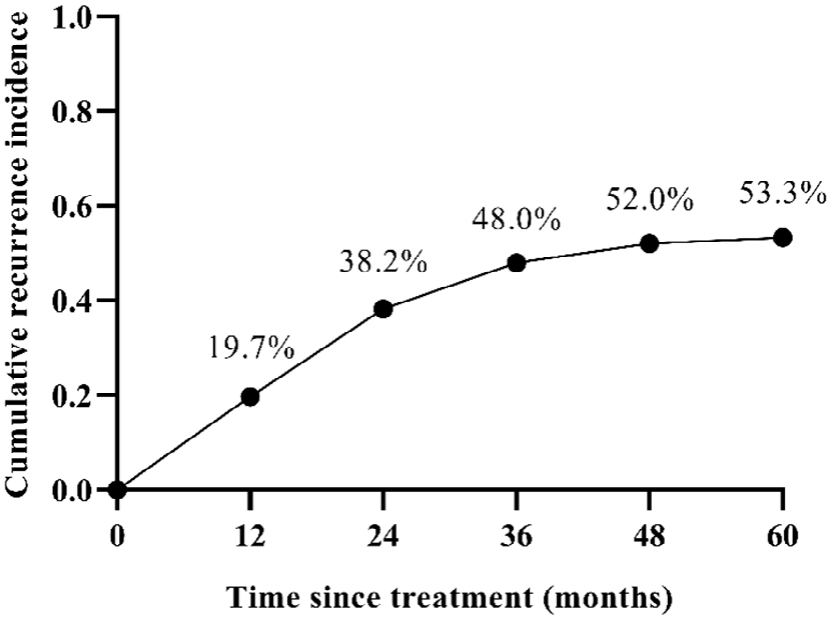
Cumulative recurrence incidence in 60 months.

The maximum follow‐up time was 74 months. HCC recurrence rates at 12, 24, 36, 48, and 60 months were 19.7% (30/152), 38.2% (58/152), 48.0% (73/152), 52.0% (79/152), and 53.3% (81/152), respectively (Figure 3). All recurrent patients had outside ptv (Planning Target Volume) recurrences and no in‐field recurrences.

In terms of overall survival situation, the 1‐, 3‐, and 5‐year overall survival rates were 92.1%, 77.3%, and 52.0%, respectively.

### 3.3 Risk Factors of HCC Recurrence

Univariate Cox regression analysis was performed for all variables, in which age (95% CI: 1.000 ~ 1.041, *p* = 0.045), 3 tumor nodules (95% CI: 3.143 ~ 217.378, *p* = 0.003), and baseline FIB‐4 (95% CI: 1.029 ~ 1.142, *p* = 0.002) were statistically different.

After considering the clinical experience and the results of univariate Cox regression, we included age, gender, tumor diameter, tumor number, and baseline FIB‐4 in a multivariate Cox regression analysis using a stepwise forward (Wald) method. The results showed that 3 tumor nodules (95% CI: 3.727 ~ 260.663, *p* = 0.002) and baseline FIB‐4 (95% CI: 1.030 ~ 1.144, *p* = 0.002) were identified as risk factors for tumor recurrence (Table 2).

**TABLE 2:** Cox regression of risk factors of HCC recurrence.

Variable	Univariate Cox regression			Multivariate Cox regression		
	HR	95%CI	*P*	HR	95%CI	*P*
Age	1.020	1.000 ~ 1.041	0.045			
Gender						
Male			Ref			
Female	−0.327	0.422 ~ 1.233	0.233			
Tumor diameter	1.229	0.999 ~ 1.513	0.051			
Tumor num						
1			Ref			Ref
2	1.728	0.751 ~ 3.977	0.198	1.618	0.703 ~ 3.726	0.258
3	26.137	3.143 ~ 217.378	0.003	31.169	3.727 ~ 260.663	0.002
BCLC						
0			Ref			
A	0.538	0.216 ~ 1.342	0.184			
Alcohol						
NO			Ref			
YES	0.649	0.089 ~ 4.723	0.649			
Child‐Pugh						
A			Ref			
B	1.962	0.945 ~ 4.076	0.071			
Cirrhosis						
0			Ref			
1	1.414	0.517 ~ 3.868	0.500			
WBC	0.987	0.864 ~ 1.128	0.847			
HGB	0.999	0.988 ~ 1.011	0.875			
Platelets	0.998	0.994 ~ 1.002	0.260			
ALT	1.000	0.994 ~ 1.005	0.951			
AST	1.004	0.999 ~ 1.008	0.086			
Albumin	0.977	0.940 ~ 1.016	0.241			
TBIL	1.007	0.998 ~ 1.016	0.116			
AFP	1.001	1.000 ~ 1.002	0.723			
FIB‐4 (baseline)	1.084	1.029 ~ 1.142	0.002	1.085	1.030 ~ 1.144	0.002
FIB‐4 (3‐month)	1.045	0.995 ~ 1.097	0.079			
FIB‐4 (6‐month)	1.045	0.991 ~ 1.101	0.106			

Abbreviations: AFP, alpha‐fetoprotein; ALT, alanine transaminase; AST, aspartate aminotransferase; BCLC, Barcelona Clinic Liver Cancer; FIB‐4, fibrosis index based on four factors; HGB, hemoglobin; TBIL, total bilirubin; WBC, white blood cells.

### 3.4 The Change of FIB‐4 Over 6 Months

**FIGURE 4:**
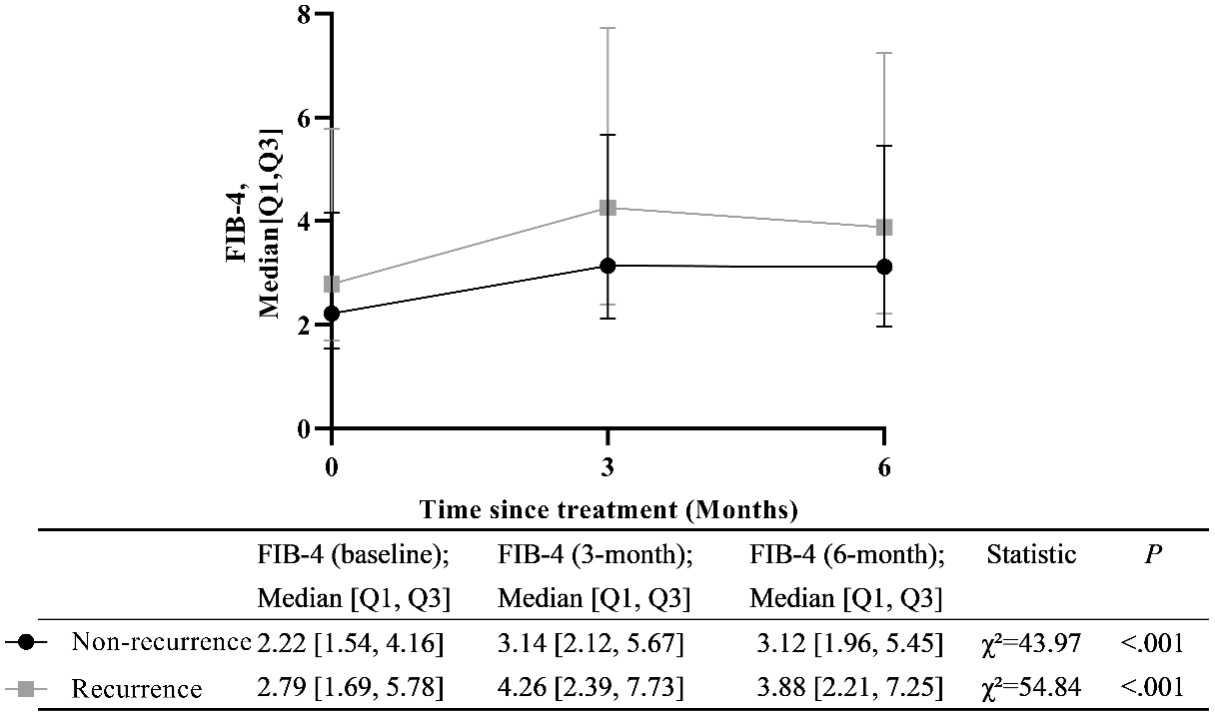
The change of FIB‐4 over 6 months. *χ* ^2^, Friedman test. FIB‐4, fibrosis index based on four factors.

In both the recurrence group and non‐recurrence group, we observed that the baseline FIB‐4 was significantly lower than those at 3 and 6 months (*P* < 0.001, Figure 4).

The difference between FIB‐4 measured at 3 months and FIB‐4 at baseline was recorded (FIB‐4 at 3 months minus FIB‐4 at baseline), and the differences between FIB‐4 at 6 months and baseline, and 6 months and 3 months were obtained in the same way. Compare the difference in FIB‐4 scores between the two groups of patients at the same follow‐up time points. The results indicated that there were no significant differences in FIB‐4 values between the two groups of patients at 3 months minus baseline (*p* = 0.806), 6 months minus baseline (*p* = 0.247), and 6 months minus 3 months (*p* = 0.257) (Table 3).

**TABLE 3:** Comparison of FIB‐4 differences between the two groups of patients with different follow‐up times.

	Non‐recurrence (*N* = 70)	Recurrence (*N* = 82)	Statistic	*p*
FIB‐4 (3 months) minus FIB‐4 (baseline)	0.96 [0.24, 1.85]^a^	1.05 [0.36, 2.11]^a^	*Z* = −0.25	0.806
FIB‐4 (6 months) minus FIB‐4 (baseline)	0.70 [0.17, 1.70]^a^	0.59 [−0.03, 1.43]^a^	*Z* = −1.16	0.247
FIB‐4 (6 months) minus FIB‐4 (3 months)	−0.11 [−0.54, 0.29]^a^	−0.21 [−0.77, 0.16]^a^	*Z* = −1.13	0.257

*Note: Z*: Mann–Whitney *U* test.

Abbreviation: FIB‐4, fibrosis index based on four factors.

^a^ M [Q1, Q3].

To further investigate the effect of FIB‐4 changes on HCC recurrence, patients were categorized into FIB‐4 falling and rising groups based on the change in the difference between baseline FIB‐4 and FIB‐4 at 3 and 6 months. Comparison of the differences in HCC recurrence between the FIB‐4 falling and rising groups over the three time periods (baseline to 3 months, baseline to 6 months, and 3 months to 6 months) showed that there was no statistically significant difference between the falling or rising in FIB‐4 and the recurrence of HCC over the three time periods (*p* = 0.385, *p* = 0.153, *p* = 0.155, respectively) (Table 4).

**TABLE 4:** Comparison of the differences in HCC recurrence between the FIB‐4 falling and rising groups over the three time periods.

		Non‐recurrence (*N* = 70)	Recurrence (*N* = 82)	Statistic	*p*
FIB‐4 (3 months) minus FIB‐4 (baseline)	Falling (*N* = 24)	13	11	*χ* ^2^ = 0.755	0.385
	Rising (*N* = 128)	57	71		
FIB‐4 (6 months) minus FIB‐4 (baseline)	Falling (*N* = 34)	12	22	*χ* ^2^ = 2.040	0.153
	Rising (*N* = 118)	58	60		
FIB‐4 (6 months) minus FIB‐4 (3 months)	Falling (*N* = 96)	40	56	*χ* ^2^ = 2.018	0.155
	Rising (*N* = 56)	30	26		

*Note: χ* ^2^: Pearson chi‐squared test.

Abbreviation: FIB‐4, fibrosis index based on four factors.

### 3.5 Correlation of Baseline FIB‐4 With Other Markers

72.9% of the patients were in the non‐recurrence group with portal hypertension, while 78.0% of the patients were in the recurrence group with portal hypertension. FIB‐4 had a positive correlation with portal hypertension (*ρ* = 0.312, *p* < 0.001).

Child‐Pugh scores were calculated at 3 and 6 months after SBRT, and the Child‐Pugh scores at the two time points were subtracted from the baseline Child‐Pugh scores, respectively, and the changes in Child‐Pugh were categorized as decrease, maintenance, and increase based on the difference. The correlation between the Child‐Pugh difference and the FIB‐4 difference at the same time point was analyzed.

The results showed that there was no correlation between Child‐Pugh scores and FIB‐4 changes at 3 months (Kendall's Tau‐b = 0.112, *p* = 0.155), but there was a positive correlation between changes at 6 months (Kendall's Tau‐b = 0.230, *p* = 0.003). This means that at 6 months after SBRT, patients with rising FIB‐4 were more likely to have rising Child‐Pugh scores as well.

### 3.6 Grading of Baseline FIB‐4

ROC was done on baseline FIB‐4 and cutoff value was calculated as 6.55 based on Jordon's index (0.201). Patients were divided into two groups by the cutoff value of 6.55. FIB4‐grade1: baseline FIB‐4 < 6.55. FIB4‐grade2: baseline FIB‐4 ≥ 6.55.

**FIGURE 5:**
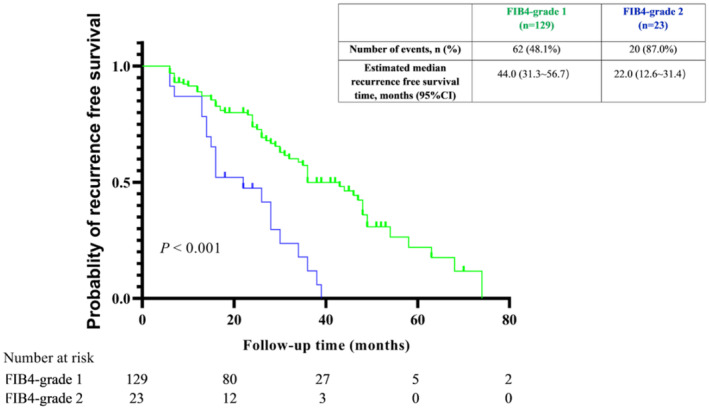
Kaplan–Meier curves according to baseline FIB‐4 grade. FIB‐4, fibrosis index based on four factors.

Based on the results of multivariate Cox regression, we conducted survival analyses of baseline FIB‐4. Baseline FIB‐4 was classified into two grades. FIB‐4 grade 1 and 2 accounted for 84.8% (129/152), and 15.2% (23/152) of the total number of patients. The RFS time of FIB‐4 grade 2 patients was significantly lower than grade 1 (*p* < 0.001, Figure 5).

## 4 Discussion

HCC is a severe disease that causes approximately 782,000 deaths per year worldwide [2]. Research on the treatment of HCC has never ceased, and to date, there are several effective treatment methods [4]. SBRT has its unique advantages, and more patients will choose it as a treatment option in the future [5]. Preventing the recurrence of HCC is a crucial aspect of its treatment. Therefore, identifying patients at high risk of recurrence is of great significance. Previous researchers have found that certain proteins or factors play a promising role in predicting HCC prognosis [6, 7, 8]. However, there are inconveniences in measuring the levels of these proteins or factors, which lead to difficulties in widespread clinical detection. A simple and accessible indicator is needed.

FIB‐4 is calculated using age, AST, ALT, and PLT, which are readily available in clinical practice. When FIB‐4 was first proposed in 2006, the indicator was expanded from its origins in HCV/HIV‐coinfected patients to encompass other liver diseases. FIB‐4 was first applied to HCC in 2011 [16]. The study in 2011 identified FIB‐4 as a risk factor for the development of HCC in HIV‐infected patients [16]. A subsequent study found that a high FIB‐4 was a risk factor for the development of HCC in patients infected with HBV [17]. The use of FIB‐4 as a predictive marker has some rational advantages. Firstly, FIB‐4 is a noninvasive test that does not cause additional harm to the patient [18]. Secondly, FIB‐4 does not incur additional expenses and thus has the advantage of being cost‐effective [19]. Thirdly, FIB‐4 is quickly and easily obtained. Based on the above information, we conducted a retrospective study to analyze the predictive role of FIB‐4 in HCC recurrence in patients with HBV‐related small HCC after SBRT.

Tsai, M. et al. conducted a retrospective study on HBV‐related HCC patients to investigate the role of FIB‐4 in predicting HCC recurrence after resection treatment [14]. Their study found that preoperative FIB‐4 and 12‐month postoperative FIB‐4 can predict the recurrence of HCC. In our study, we also found that the baseline FIB‐4 has predictive value for recurrence in patients with HBV‐related HCC after SBRT. However, we did not find that changes in FIB‐4 were useful in predicting the recurrence of HCC at 3 or 6 months after SBRT. Our study did not demonstrate a role for FIB‐4 at 12 months. We began evaluating the recurrence in follow‐up patients at 6 months, and it was during the 6‐month assessment that the earliest cases of recurrence were identified.

In the comparison of the two groups of patients, the baseline FIB‐4 was higher in the recurrence group than in the non‐recurrence group. However, after treatment, both groups exhibited an increase in FIB‐4, which was statistically significant compared to the baseline values. FIB‐4 is an indicator of the degree of liver fibrosis, where a higher FIB‐4 value indicates more severe fibrosis. But FIB‐4 is not directly measured, but calculated. This means that FIB‐4 is not an absolute reflection of the degree of fibrosis in the liver and can also be affected by factors such as liver inflammation, bone marrow depression, etc. The patients in this study were treated with radiotherapy, which may cause a decrease in platelets. On the one hand, radiotherapy may lead to bone marrow suppression and stem cell damage, resulting in reduced platelet production. On the other hand, radiotherapy may also directly damage platelets, leading to a shortened lifespan. Magne N. et al. found that radiotherapy can cause a decrease in platelets that lasts for up to 6 months [20]. Platelets are included in the formula for FIB‐4, this is the reason for the elevation of FIB‐4 after treatment observed in the present study, and may partly explain the inability of postoperative FIB‐4 to predict HCC recurrence.

Liao. R. et al. used 3.25 as the FIB‐4 cut‐off value and Tsai, M. et al. used 2 as the cut‐off value for their study [13, 14]. In our study, we stratified the study patients using a cut‐off value of 6.55 and then conducted survival analysis. The results of the analysis were statistically significant. The reason for our FIB‐4 values being higher than previous studies is that 94.06% of the study patients had liver cirrhosis, and the presence of cirrhosis made the median baseline FIB‐4 higher. This implies that using FIB‐4 to predict HCC recurrence in patients with liver cirrhosis needs to be considered with a higher FIB‐4 cut‐off value.

In this study, three tumor nodules were also found to be a risk factor for HCC recurrence in multivariate Cox regression. However, its high hazard ratio (HR) and wide confidence interval suggest that this result is mostly affected by the small sample size and uneven frequency distribution. In fact, there was only one patient with three tumor nodules. Nevertheless, it is still possible that the number of tumor nodules may have an impact on tumor recurrence. Study from Lin et al. [21] found that tumor recurrence is associated with the number of tumors present before treatment. Another previous study has shown that early recurrence (recurrence time < 2 years) of HCC is related to the attributes of the tumor, while late recurrence (recurrence time > 2 years) is associated with chronic liver disease [22]. In our study, 38.2% of patients experienced early recurrence, while 15.7% experienced late recurrence. This also implies that the number of tumor nodules is a factor affecting prognosis in our study population. Nevertheless, more larger sample studies are needed to prove this conclusion.

This study consecutively observed HCC recurrence in patients with HBV‐related HCC after SBRT and also investigated the role of FIB‐4 in predicting recurrence in these patients. No previous studies have been conducted in this area. However, our study has some shortcomings. Firstly, the sample size is not large enough. Secondly, we did not establish a validation cohort due to the limitation in sample size. Furthermore, our study population consisted of Chinese individuals and was limited to HBV infection. It is unclear whether the conclusions are applicable to other national and regional populations, or to patients with hepatitis C, alcoholic hepatitis, or fatty liver HCC. In the future, prospective studies could be designed to include a greater number and diversity of patients for in‐depth study.

In our study, we found that the baseline FIB‐4 could play a role in predicting recurrence in HBV‐related HCC patients after SBRT. Patients with liver cirrhosis who had a baseline FIB‐4 higher than 6.55 were more likely to experience HCC recurrence compared to those with a baseline FIB‐4 score lower than 6.55. The change in FIB‐4 after SBRT was not relevant for predicting HCC recurrence. Our study can contribute to the clinical follow‐up monitoring of postoperative patients with HCC. This can aid in identifying patients at high risk of recurrence, enabling more rational observation and adjuvant therapy.

## Author Contributions

**Zhan Zeng:** conceptualization (equal), formal analysis (equal), methodology (equal), writing – original draft (lead). **Yifan Han:** conceptualization (equal), formal analysis (equal), methodology (equal), writing – review and editing (lead). **Hongyu Chen:** investigation (equal). **Ning Lin:** investigation (equal). **Xiaoyuan Xu:** funding acquisition (lead), supervision (lead). **Yanyan Yu:** supervision (equal). **Wengang Li:** resources (lead).

## Ethics Statement

This study was approved by the Ethics Committee of the Fifth Medical Center of the Chinese People's Liberation Army General Hospital (procedure code 2020‐063‐D).

## Consent

All patients gave informed consent.

## Conflicts of Interest

The authors declare no conflicts of interest.

## Data Availability

The data that support the findings of this study are available on request from the corresponding author. The data are not publicly available due to privacy or ethical restrictions.
